# Modulation of intratumoural myeloid cells, the hallmark of the anti-tumour efficacy induced by a triple combination: tumour-associated peptide, TLR-3 ligand and α-PD-1

**DOI:** 10.1038/s41416-020-01239-z

**Published:** 2021-02-03

**Authors:** Sara Zalba, Virginia Belsúe, Brian Topp, Dinesh de Alwis, Maite Alvarez, Iñaki F. Trocóniz, Pedro Berraondo, María J. Garrido

**Affiliations:** 1 Department of Pharmaceutical Technology and Chemistry, Faculty of Pharmacy and Nutrition, University of Navarra, Pamplona, Spain; 2 Navarra Institute for Health Research (IdiSNA), Pamplona, Spain; 3 Program of Immunology and Immunotherapy, CIMA Universidad de Navarra, Pamplona, Spain; 4 Merck & Co., Inc, Kenilworth, NJ USA; 5 Centro de Investigación Biomédica en Red de Cáncer (CIBERONC), Madrid, Spain

**Keywords:** Oncology, Cancer

## Abstract

**Background:**

Anti-programmed cell death 1 (PD-1)/programmed death-ligand 1 (PD-L1) monoclonal antibodies (mAbs) show remarkable clinical anti-tumour efficacy. However, rational combinations are needed to extend the clinical benefit to primary resistant tumours. The design of such combinations requires the identification of the kinetics of critical immune cell populations in the tumour microenvironment.

**Methods:**

In this study, we compared the kinetics of immune cells in the tumour microenvironment upon treatment with immunotherapy combinations with different anti-tumour efficacies in the non-inflamed tumour model TC-1/A9. Tumour-bearing C57BL/6J mice were treated with all possible combinations of a human papillomavirus (HPV) E7 long peptide, polyinosinic–polycytidylic acid (PIC) and anti-PD-1 mAb. Tumour growth and kinetics of the relevant immune cell populations were assessed over time. The involvement of key immune cells was confirmed by depletion with mAbs and immunophenotyping with multiparametric flow cytometry.

**Results:**

The maximum anti-tumour efficacy was achieved after intratumoural administration of HPV E7 long peptide and PIC combined with the systemic administration of anti-PD-1 mAb. The intratumoural immune cell kinetics of this combination was characterised by a biphasic immune response. An initial upsurge of proinflammatory myeloid cells led to a further rise in effector CD8^+^ T lymphocytes at day 8. Depletion of either myeloid cells or CD8^+^ T lymphocytes diminished the anti-tumour efficacy of the combination.

**Conclusions:**

The anti-tumour efficacy of a successful immunotherapy combination in a non-inflamed tumour model relies on an early inflammatory process that remodels the myeloid cell compartment.

## Background

Immunotherapy is currently one of the most important and promising therapeutic mainstays of oncology. This relevance relies on the ability to modulate the immune system to eliminate cancer cells.^1^

However, patient stratification and personalised treatments are required to get the most from this cancer treatment strategy.^2^ For this purpose, the immune baseline status of tumours, regarding certain markers such as T cell infiltration, chemokine profiles, major histocompatibility complex (MHC) class I, and programmed death-ligand 1 (PD-L1) expression, is crucial.^2^ Indeed, these specific characteristics have been used for tumour classification into inflamed or hot tumours, and non-inflamed or cold tumours. Inflamed tumours are characterised by an abundant infiltration of effector immune cells and are sensitive to immune checkpoint (IC) inhibitors. In contrast, non-inflamed tumours, which are more resistant to immunotherapy, present low T cell infiltration and a paucity of MHC class I and PD-L1 expression, thus favouring tumour escape.^3^ For these cold tumours, approaches such as vaccination, radiotherapy, chemotherapy or adoptive cellular therapy need to be combined in order to obtain a clinical response.^4^ Indeed, the main challenge for immunotherapy is to switch the tumour phenotype from cold to hot using appropriate treatments. In that sense, according to the cancer-immunity cycle reported by Chen and Mellman,^5^ several specific processes, such as activation of antigen-presenting cells (APCs), abundant tumour-infiltrating T cells and presence of interferon-α (IFN-α)/interleukin-12 (IL-12), are of particular importance to achieve effective tumour regression.^6–9^ Therapeutic cancer vaccines can promote these processes, eliciting a potent T cell-mediated immune response able to recognise and eliminate cancer cells expressing specific tumour-associated antigens.^10^ The success of therapeutic cancer vaccines relies on the choice of an adjuvant to ensure the proper stimulation of innate immunity. Among the different adjuvants, Toll-like receptor (TLR) agonists are often used in cancer immunotherapy.^11–13^ Thus, polyinosinic–polycytidylic acid (PIC), a TLR-3 agonist, is able to induce proinflammatory immune signalling that leads to tumour regression.^12–15^ Recently, the intratumoural (i.t.) injection of cancer vaccines has been proposed as an alternative therapeutic strategy able to induce a potent anti-tumour effect.^16–18^ However, clinical results have shown that therapeutic cancer vaccines are unable to attain significant effects on overall survival. To escape from the control of anti-tumour immune responses elicited by vaccines, tumours have developed multiple immune-resistance mechanisms such as over-expression of ICs, including the programmed cell death-1 (PD-1)/PD-L1 axis.^10,19^ Blockade of this axis using anti-PD-1/PD-L1 monoclonal antibodies (mAbs) has demonstrated unprecedented clinical benefit enhancing patient outcomes and shifting the equilibrium against tumour tolerance.^20,21^

The design of clinical trials of rational combinations must be based on a profound understanding of the critical immune cell populations and the intratumoural kinetics of these cells. Unfortunately, exhaustive information about how these critical populations fluctuate over time in the tumour microenvironment is scarce. Longitudinal data collection in immunocompetent mouse models can provide an adequate characterisation of the relevant events occurring before and during immunotherapy treatment.^22^ Thus, this study aims to compare the differences in the intratumoural kinetics of critical immune cell subsets subjected to immunotherapy combinations with a range of anti-tumour effects in a poorly immunogenic mouse tumour model.

## Methods

### Materials

The TLR-3 agonist PIC, IFNγ and EDTA 0.5 M (pH 8) were obtained from Thermo Fisher (Massachusetts, USA). The synthetic peptide DWLKYKDKLKEKLKEALFPDWLKYKDKRAHYNIVTFF that contains the human papillomavirus 16 (HPV16) E7 H2-Db-restricted epitope E7 (49–57) was synthesised by GenScript (New Jersey, USA). Rat anti-mouse α-PD-1 (CD279; clone RMP1–14), anti-CD8α (clone 56-6.7), anti-GR-1 (clone RB6-8C5) and polyclonal rat anti-IgG2 antibody were purchased from BioXCell (New Hampshire, USA). Tween-20, streptavidin−peroxidase, Percoll and bovine serum albumin were bought from Sigma-Aldrich (Missouri, USA). EDTA tubes were obtained from Stardstedt (Nümbrecht, Germany). Collagenase D and DNAase I were from Roche (Basel, Switzerland). ACK (ammonium-chloride-potassium) buffer was purchased from Lonza (Basel, Switzerland). Ninety-six V-shaped wells were bought from Greiner Bio-One (Kremsmünster, Austria), 6-well plates were from Corning (New York, USA) and 70 µm cell strainers were obtained from BD Falcon (New Jersey, USA).

### Flow cytometry and immunohistochemistry

Flow cytometry analyses were performed with FACSCalibur^TM^ or CytoFLEX Flow Cytometers (BD Biosciences, California, USA) and data processed with FlowJo^TM^10.6.2 (Oregon, USA). Zombie Nir^TM^ Kit was obtained from BioLegend® (California, USA) and Mouse Regulatory T Cell Staining Kit was from eBioscience (California, USA). The rest of the used antibodies are listed in Table S1.

### Cell line and tumour model

All experiments were carried out using the murine TC-1/A9 cell line. This cell line, derived from primary mouse lung epithelial cells, expresses the E7 protein from HPV constitutively and is characterised by low MHC class I expression.^12^

Cells were maintained in RPMI-1640 with GlutaMAX supplemented with foetal bovine serum [10% (v/v)], penicillin/streptomycin [1% (v/v)], Geneticin (0.4 mg/ml) and 2-mercaptoethanol (0.05 mM), all obtained from Gibco (California, USA). Growth conditions were 5% of CO_2_ and 37 °C in a water-saturated atmosphere. Cells were split twice per week when a confluence of 60–70% was reached. Mycoplasma was regularly tested with a luminescence assay (Lonza, Basel, Switzerland).

Five-week-old female immunocompetent C57BL/6J mice supplied by Harlan (Barcelona, Spain) were housed in sterile plastic cages with enrichment elements and wood chips as bedding material. A maximum of six animals were allocated to each cage and were kept under standard and sterile conditions (25 °C, 50% relative humidity, 12 h dark/light) with sterile water and standard food ad libitum, at the animal facility of the University of Navarra. Tumour cells (1 × 10^5^) were inoculated subcutaneously in 100 µl of phosphate-buffered saline (PBS) (Gibco) on the right flank of the animal. Seven days later, when the average tumour diameter reached 5 mm, mice were randomly divided into the corresponding groups to receive each treatment. At the end of the experiment or according to the end-point criteria established by the protocol ref. 023-17, mice were sacrificed using CO_2_.

### Evaluation of immunological characteristics of the TC-1/A9 tumour

PD-L1 expression measured in TC-1/A9 culture cells and tumour-infiltrating lymphocytes (TILs) characterised ex vivo were determined to characterise the baseline immunological status of the present tumour model.

Thus, to determine PD-L1 expression, 1 × 10^5^ TC-1/A9 cells per well were seeded into 6-well plates in 3 ml of medium. After 24 h, cells were collected into 96-well V-shaped cell plates and incubated 15 min at 4 °C with the rat anti-PD-L1 in PBS, followed by a stain with anti-rat-IgG-AF488 at room temperature (RT) for 15 min. In a parallel plate, PD-L1 expression was induced by 48 h exposure to IFNγ (20 ng/ml).^23^ These cells were washed thoroughly and stained for flow cytometry analysis.

For TIL evaluation, tumours were collected from non-treated mice sacrificed at day 10 after TC-1/A9 cell inoculation. Tumours were kept in dry ice and immediately preserved at −80 °C embedded in OCT gel. The frozen tissue was cut into 5 µm slices and stained for CD3 and CD8. Images were scanned with the Aperio CS2 (Leica Biosystems, Wetzlar, Germany).

### Efficacy evaluation with different combinations

The anti-tumour efficacy was assayed in TC-1/A9 tumour-bearing mice treated with different combinations. Female mice were randomly divided into eight groups: control; monotherapies: E7 long peptide; PIC; anti-PD-1; bitherapies: E7 long peptide/PIC; E7 long peptide/anti-PD-1; PIC/anti-PD-1; triple therapy: E7 long peptide/PIC/anti-PD-1. The E7 long peptide (100 µg/mouse) and PIC (50 µg/mouse) were administered i.t. at days 7 and 14. For i.t. administration, 100 µl were slowly injected into the tumour mass at an angle of 45°. Anti-PD-1 (200 µg/mouse) was prepared in 100 µl of PBS and injected intravenously as a bolus at days 7, 10, 14 and 17, after tumour implantation.

Each group received the corresponding treatment following the experimental schedule represented in Fig. S1a, b. Tumour size was measured twice a week using an electronic calliper and was expressed as mean diameter (MD) calculated by the formula:

$${\mathrm{MD}} = \left( {{L} + {W}} \right)/2,$$

where *L* represents the length and *W* the width of the tumour.

Tumour growth and body weight were monitored twice weekly until the maximum tumour size was reached, and the mice were then sacrificed. In the case of tumour shrinkage, animals were monitored for 3 months to check for possible tumour relapse. Other possible signs of toxicity, such as ulceration, were carefully recorded to ensure mouse welfare. In order to explore the time evolution of the different treatments, the RECIST 1.1. criteria^24^ were applied, taking the tumour size at day 7 after cell inoculation, as the baseline for comparisons. Complete response (CR) corresponded to a tumour size reduction of 100% compared to baseline; partial response (PR), the reduction was >50%; stable disease (SD), tumour size ranged between a reduction <50% and an increment <25% compared to baseline; finally, progressive disease was associated with an increase of the baseline >25%.

### Biomarkers associated with the immune response

Female TC-1/A9 tumour-bearing mice were divided into eight groups, using the immunotherapy combinations described above. At different time points: 3, 8 and 13 days after treatment administration, three animals per group were sacrificed for tumour collection, as shown in Fig. S2.

Tumour tissue was digested with 5 ml of collagenase/DNase I (1:10) for 30 min at 37 °C. The reaction was stopped by adding 50 µl of EDTA (0.5 M). Cells were forced through 70 µm cell strainers, centrifuged and incubated with 1 ml of ACK buffer for 1 min at RT. Pellets were collected by centrifugation at 600 × *g* for 3 min and transferred to a V-shaped plate for the staining of CD45, CD3/TCRβ, CD8, CD11b, LY6C, PD-L1 and CD4, CD25 and Foxp3 (Table S1). These samples were measured immediately by flow cytometry.

In addition, an extra group of TC-1/A9 tumour-bearing mice injected with the triple combination was used to evaluate TILs at days 3 and 8 after treatment. Three mice at each time were sacrificed, and tumours were excised and frozen in OCT for CD3^+^ and CD8^+^ staining.

### Myeloid and CD8^+^ T cell depletion

The role of myeloid and CD8^+^ T cells in the anti-tumour effect of the triple therapy was investigated. Thus, at day 7 after TC-1/A9 cell inoculation, mice were divided into three groups: control and triple therapy combined with anti-GR-1 antibody or with an irrelevant antibody. The GR-1 antibody is reported to bind specifically to immature myeloid cells, leading to their depletion,^25^ whereas the irrelevant polyclonal rat α-IgG2 was administered as an antibody control. Anti-GR-1 and anti-IgG2 were i.p. administered at 200 µg/mouse, 24 h after treatment, at days 8 and 15. Tumour size, measured twice a week and the survival rate was used to evaluate the effect of the depletion.

To evaluate the role of CD8^+^ T cells on the anti-tumour effect of the triple therapy, the following experiment was carried out. At day 7 after TC-1/A9 cell inoculation, mice were divided into three groups: control, triple therapy combined with anti-CD8 antibody and triple therapy combined with the irrelevant polyclonal rat anti-IgG2.

Doses of 200 µg/mouse of anti-CD8 were i.p. administered 24 h before therapeutic treatments and at days 2, 6, 9 and 13 after treatment, whereas anti-IgG2 was injected 24 h after the triple combination injection, at days 8 and 15. Tumour size, measured twice a week, and survival rate were used to evaluate the effect of the depletion. CD8^+^ T lymphocyte depletion was confirmed in blood 24 h after anti-CD8 mAb at days 9 and 16 by flow cytometry (Fig. S3).

CD8^+^ T cells in the tumours were characterised 24 h and 3 and 13 days after treatment administration in key treatment groups. TC-1/A9 tumour-bearing mice were divided into four groups: control, peptide, bitherapy, peptide/PIC and triple therapy: peptide/PIC/anti-PD-1. The route of administration and dose regimens were fixed according to the efficacy experiment for these groups. At selected time points, tumours from five animals per group were collected and processed.

Tumour tissue was cut into small sections and then digested with 5 ml of collagenase/DNase I for 30 min at 37 °C. The reaction was stopped by adding 50 µl of EDTA, and cells were forced through cell strainers, centrifuged and washed with 10 ml of Percoll 40% at 2000 r.p.m. for 20 min. Afterwards, cells were washed with PBS and transferred to a V-shaped plate for 10 min incubation at RT with E7 tetramer-PE to detect antigen-specific CD8^+^ cells, followed by another 10 min incubation with Zombie Nir^TM^ antibody. Cells were stained for CD45, TCRβ, CD19, PD-1, Tim3 and CD8. Samples were immediately measured by flow cytometry.

### Myeloid cell immunophenotyping in the tumour microenvironment

Based on the depletion assay with α-GR-1, the key groups of the study were selected for an in-depth analysis of the role of myeloid cells in the response. To that end, TC-1/A9 tumour-bearing mice were divided into four groups: control, peptide, peptide/PIC, and triple therapy. One day after treatment administration, mice were sacrificed, and tumours were collected for the analysis of the different myeloid subsets.

Tumour tissue was digested with collagenase/DNase I for 30 min at 37 °C. The reaction was stopped by adding 50 µl of EDTA. Cells were forced through a 70 µm cell strainer and washed with 10 ml of Percoll 40% at 2000 r.p.m. for 20 min. Pellets were washed with PBS and transferred to a V-shaped plate for 10 min incubation at RT with Zombie Nir^TM^ and stained for Ly6C, F4/80, MHCII, CD8, CD45, Ly6G, CD11c, CD19, TCRβ, CD11b, CD4, PD-L1, CD124 and CD38. Stained samples were immediately measured by flow cytometry.

### Statistical analysis

All experimental data were included for the statistical analysis that was performed using GraphPad Prism 5 (GraphPad Software, San Diego, USA). Groups were compared according to the type of treatment and the sampling times applying a one-way analysis of variance (ANOVA) or two-way ANOVA test, followed by multiple comparisons with the Tukey’s test. Statistical analysis of survival data was performed using RStudio (version 3.6.3) with the Survfit function from the survival package that computes the Kaplan–Meier estimator for truncated and/or censored data. The log-rank test was used to compare the different groups of treatments. Statistical significance was set to 0.05.

## Results

### PD-L1 expression on TC-1/A9 cells

**Fig. 1:**
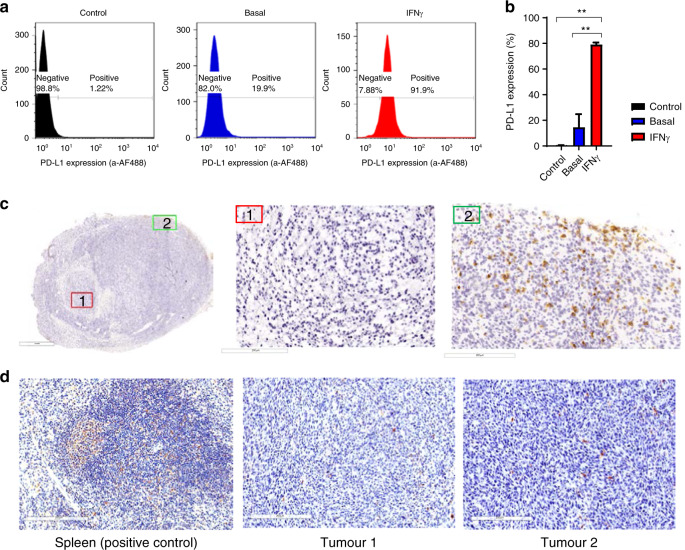
In vitro and in vivo evaluation of the immunogenicity of the TC-1/A9 tumour model. **a** Representative histograms of baseline PD-L1 expression on in vitro cultured TC-1/A9 cells and induced PD-L1 expression after 48 h of IFNγ exposure (20 ng/ml). **b** Quantification of baseline and IFNγ-induced PD-L1 expression. Pooled data from three independent experiments (*n* = 9). Data are represented as means and SD. One-way ANOVA followed by Tukey’s multiple tests. **P < 0.01. **c** Tumour tissue section (×5) from non-treated TC-1/A9 tumour-bearing mouse stained for CD3 T lymphocytes. An image with higher magnification (×20) of the tumour margin (1) and tumour centre (2) is shown. **d** PD-L1 staining of the spleen (positive control) and tumour tissue sections (×20) from a non-treated TC-1/A9 tumour-bearing mouse.

PD-L1 is the best clinically available biomarker to predict the response to anti-PD-1/PD-L1 mAbs. Thus, we characterised the PD-L1 expression on the TC-1/A9 cell line. Flow cytometry analysis showed a low baseline expression of PD-L1 with <10% of cells being positive (Fig. 1a). However, the expression was highly inducible upon IFNγ treatment. Forty-eight hours after IFNγ exposure, 80% of TC-1/A9 cells were positive for PD-L1 (Fig. 1b).

Next, we assessed the TILs and PD-L1 levels and distribution in tumour sections. Immunohistochemistry analysis revealed very low TIL infiltration, mostly at the tumour margin (Fig. 1c). In line with the in vitro PD-L1 expression and the paucity of TILs, PD-L1 expression was low (Fig. 1d). Taken together, these data support the notion of the TC-1/A9 tumour as a non-inflamed or cold tumour model.

### Immunotherapy combinations with different response rates

**Fig. 2:**
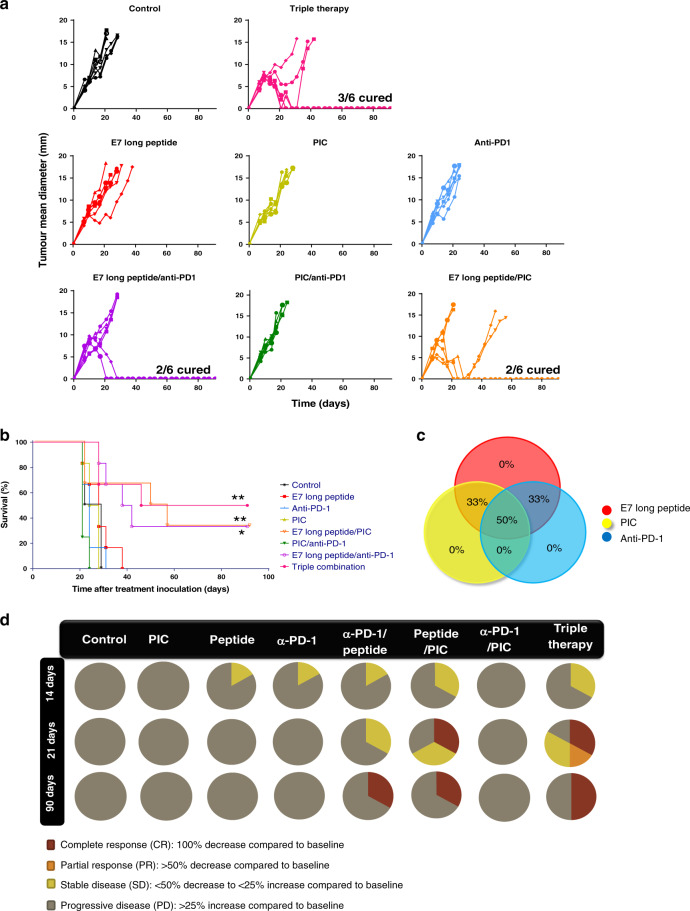
Anti-tumour efficacy of the different treatments administered to TC-1/A9 tumour-bearing mice as monotherapy or combined regimens. C57BL/6 mice were inoculated with 1 × 10^5^ TC-1/A9 cells, and 7 days later, mice were divided into the following experimental groups: control; monotherapies: E7 long peptide; PIC; anti-PD-1; bitherapies: E7 long peptide/PIC; E7 long peptide/anti-PD-1; PIC/anti-PD-1; triple therapy: E7 long peptide/PIC/anti-PD-1. The E7 long peptide (100 µg/mouse) and PIC (50 µg/mouse) were administered intratumourally (i.t.) at days 7 and 14. Anti-PD-1 (200 µg/mouse) was injected intravenously as a bolus at days 7, 10, 14 and 17, after tumour implantation. Tumour size was measured twice per week with an electronic calliper. **a** Representative profiles of tumour growth over 90 days (*n* = 6 mice/group). **b** Overall survival in the different treatment groups; differences were explored with the log-rank test. *p < 0.05 and **p < 0.01 vs. control. **c** Venn diagram representing the percentage of complete tumour regression in each experimental group. Each coloured circle represents a monotherapy treatment, whereas circle intersections represent the corresponding combination treatments with the corresponding percentage of cured animals. **d** Analysis of the anti-tumour response achieved at different times during treatments according to the RECIST 1.1 criteria. Peptide E7 long peptide, α-PD-1 anti-PD-1, Tritherapy triple combination of E7 long peptide, PIC and anti-PD-1. The data shown are representative of two independent experiments.

Next, we developed different immunotherapy combinations with a wide range of response rates to set up an experimental model that allowed us to identify the immune response that characterises successful immunotherapy. The immunotherapies tested were combinations of an E7 long peptide, PIC and anti-PD-1. Figure 2a shows tumour growth after treatment in different groups. Monotherapies (E7 long peptide, PIC or anti-PD-1 alone) did not induce any anti-tumour effect in comparison with the untreated control group. Two-to-two combinations (bitherapies) increased the response rate to 33% in the case of E7 long peptide/PIC and E7 long peptide/anti-PD-1 when compared to monotherapies (Fig. 2a, b). However, the maximum anti-tumour efficacy was achieved when combining the E7 long peptide, PIC and anti-PD-1, in which case CR was attained in 50% of cases (Fig. 2a, b). Thus, the different combinations of three immunostimulatory compounds provided an experimental setting with different response rates (summarised in Fig. 2c, d), which could provide an insight into how a cold tumour could become a hot tumour by an immunotherapeutic intervention. At day 14, just after finishing the different treatments, anti-PD-1 monotherapy and the E7 long peptide alone or in combination led to SD in 16–33% of mice. However, this therapeutic effect was transitory for monotherapies, and only E7 long peptide/anti-PD-1 and the triple combination were able to induce CRs by day 21. The triple combination was the most efficient combination, causing CR in 50% of mice at day 90 vs. 33% for the bitherapy groups. Interestingly, the in situ cancer vaccine yielded a similar response to triple therapy at early times up to day 21.

### Longitudinal analysis of the immune response in the tumour microenvironment

**Fig. 3:**
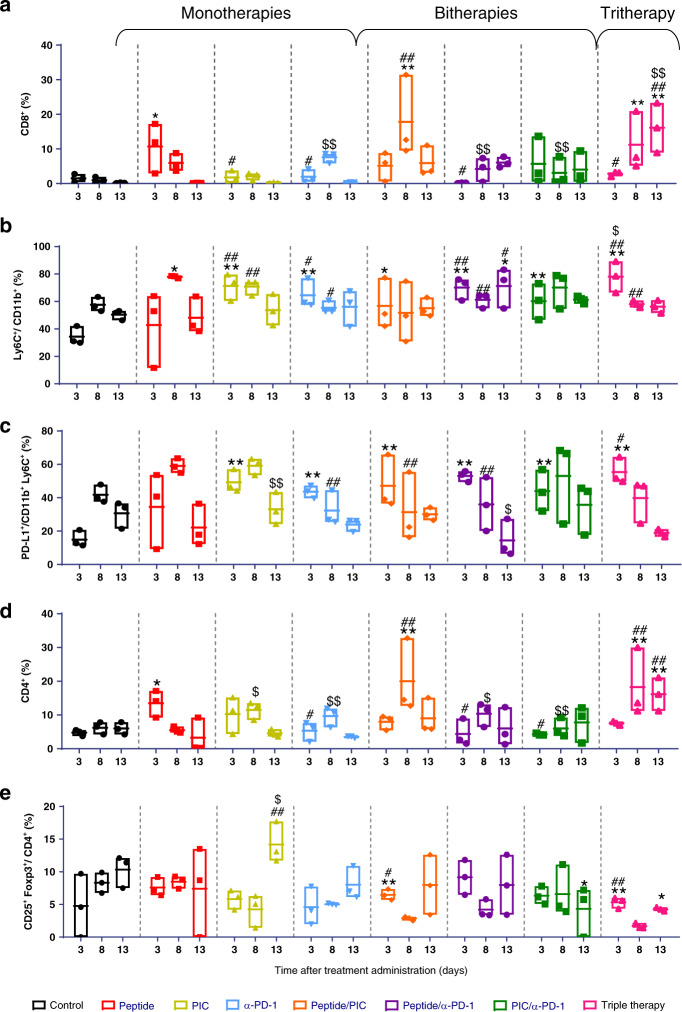
Kinetics of different immune cell populations in the tumour microenvironment after treatment administration. C57BL/6 mice were inoculated with 1 × 10^5^ TC-1/A9 cells and divided into eight groups. One week after tumour implantation mice were treated as in Fig. S1. At days 3, 8 and 13 after treatment, subgroups of three mice were sacrificed as shown in Fig. S3, and immune cells infiltrating the tumour were evaluated by flow cytometry. **a** % CD8^+^ lymphocytes in alive cells. **b** % Ly6C in CD11b^+^ myeloid cells. **c** % PD-L1 in CD11b^+^ myeloid cells. **d** % CD4^+^ lymphocytes in alive cells. **e** CD25^+^ Foxp3^+^ in CD4^+^ (*n* = 3). Two-way ANOVA followed by multiple comparisons (Tukey’s test): *p < 0.05 vs. control; **p < 0.01 vs. control; ^#^*p* < 0.05 vs. peptide, ^##^*p* < 0.05 vs. peptide; ^$^*p* < 0.05 vs. peptide/PIC, ^$$^*p* < 0.01 vs. peptide/PIC. Peptide E7 long peptide, α-PD-1 anti-PD-1, Tritherapy: triple combination of E7 long peptide, PIC and anti-PD-1.

In order to identify the immune response that conveys maximum anti-tumour activity, we analysed the kinetics of different immune cells in the tumour microenvironment upon different treatments. Figure 3 shows the time evolution of CD8^+^ T lymphocytes (Fig. 3a), Ly6C and PD-L1 expression on CD11b^+^ myeloid cells (Fig. 3b, c), CD4^+^ T lymphocytes (Fig. 3d) and T-regulatory (Treg) cells (Fig. 3e). The immune response upon treatment with the E7 long peptide alone was characterised by an increase in CD8^+^ T cells at day 3 and an increase of PD-L1 expression in monocytes on day 8. However, these effects were rapidly downregulated, leading to a significant increase in Tregs on day 13. This was also the most prominent effect of PIC in monotherapy, while the anti-PD-1-alone activity was reflected in a peak expression of PD-L1 in myeloid cells on day 3.

Two-to-two combinations exerted a more profound modulation of immune cells in the tumour microenvironment. The three groups displayed a significant increase in Ly6C^+^ and PD-L1 in CD11b^+^ myeloid cells on day 3, suggesting an initial proinflammatory process. Only the combination of E7 long peptide/PIC induced an increase in CD8^+^ T lymphocytes and CD4^+^ T lymphocytes at day 8, but this effect was transient and returned to baseline levels at day 13 (Fig. 3a). In contrast, triple therapy achieved a sustained increase in CD8^+^ and CD4^+^ T lymphocytes at days 8 and 13. Despite the high increase of CD4^+^ T cells, Treg decreased significantly (Fig. 3e). The modulation of T lymphocytes was preceded by the highest levels of Ly6C and PD-L1 on CD11b^+^ myeloid cells.

Interestingly, those combinations achieving anti-tumour efficacy induced immune memory in those mice that completely eradicated the tumour because tumour cells were rejected after a re-challenge (Fig. S4).

### Myeloid cell depletion reduces triple therapy efficacy mediated by CD8^+^ T lymphocytes

Based on the strong modulation of myeloid cells and CD8^+^ T lymphocytes mediated by the triple therapy, we analysed the role of these cells using depleting mAbs.

**Fig. 4:**
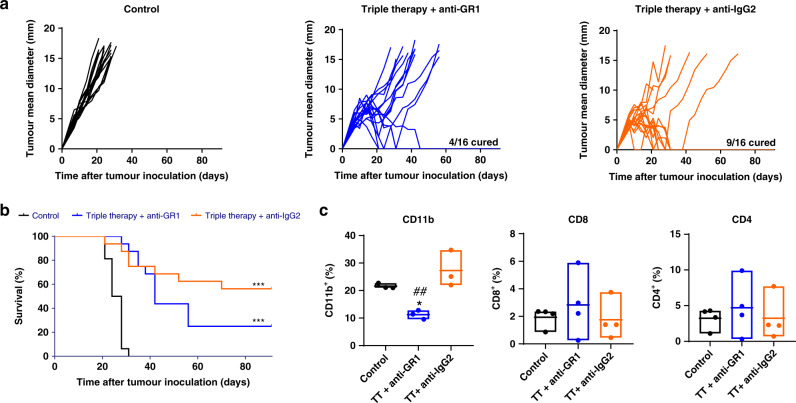
Effect of myeloid cells on the anti-tumour activity of the triple therapy. C57BL/6 mice were inoculated with 1 × 10^5^ TC-1/A9 cells, and 7 days later, mice were divided into three groups: control and triple therapy combined with anti-GR-1 antibody or triple therapy combined with an irrelevant antibody. Anti-GR-1 and anti-IgG2 were i.p. administered at 200 µg/mouse, 24 h after treatment and at days 8 and 15. Tumour size was measured twice a week with an electronic calliper. **a** Tumour growth profiles over 90 days. Pooled data from two independent experiments (*n* = 16/group). **b** Overall survival of the different treatment groups. Pooled data from two independent experiments (*n* = 16/group). Log-rank test. ***p < 0.001 vs. control. **c** Percentage of different immune populations in tumour tissue 24 h after α-GR-1 administration (*n* = 5/group). One-way ANOVA followed by Tukey’s multiple comparisons. *p < 0.05 vs. control, ^##^*p* < 0.01 vs. TT + anti-IgG2. TT triple therapy.

Administration of anti-GR-1 mAb 24 h after initiation of triple therapy reduced the anti-tumour efficacy (Fig. 4a, b). The anti-tumour efficacy following myeloid cell depletion was similar to the effect achieved with the combined treatment with E7 long peptide/PIC or E7 long peptide/anti-PD-1. We confirmed that anti-GR-1 mAb administration reduced the percentage of CD11b^+^ myeloid cells in the tumour microenvironment with no effect on CD8^+^ or CD4^+^ T lymphocytes (Fig. 4c).

**Fig. 5:**
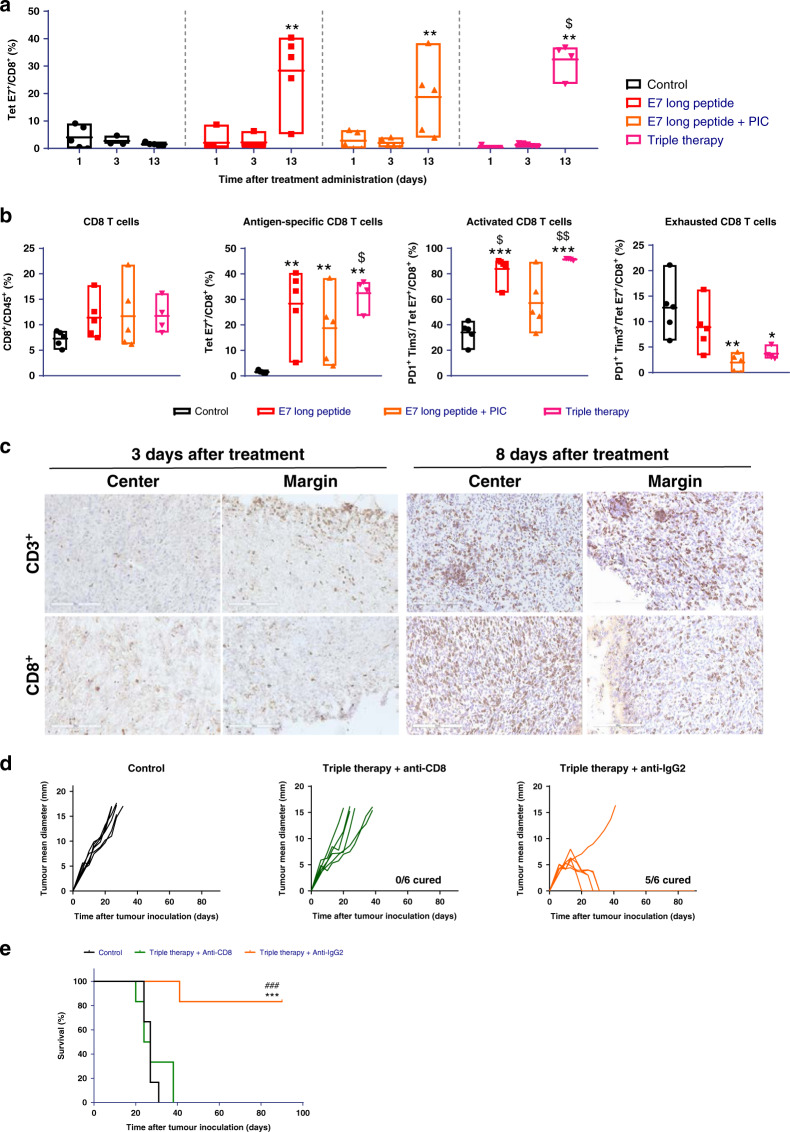
Evaluation of CD8^+^ T lymphocytes anti-tumour activity of the triple therapy-treated mice. **a** C57BL/6 mice were inoculated with 1 × 10^5^ TC-1/A9 cells, and 7 days later, they were treated with an E7 long peptide (100 µg/mouse, i.t.), E7 long peptide with PIC (50 µg/mouse, i.t.) or the triple therapy. At days 1, 3 and 13 after treatment, E7-specific CD8^+^ T-cells were analysed on tumour tissue; **b** additionally, at day 13, CD8^+^ subpopulations were also analyzed on these tumour tissues (*n* = 5/group). Two-way-ANOVA followed by multiple comparisons (Tukey’s test). *p < 0.05 and **p < 0.01 vs. control; ^$^*p* < 0.05 and ^$$^*p* < 0.01 vs. E7 long peptide/PIC. **c** detection of CD3^+^ and CD8^+^ T lymphocytes by immunohistochemistry on TC-1/A9 tumours collected at days 3 and 8 after triple therapy administration (bar corresponds to 200 μm or 20x). **d** At day 7 after 1 × 10^5^ TC-1/A9 cell inoculation, mice were divided into three groups: control, triple therapy combined with anti-CD8 antibody and triple therapy combined with the irrelevant polyclonal rat anti-IgG2. Doses of 200 µg/mouse of anti-CD8 were i.p. administered 24 h before therapeutic treatments and at days 2, 6, 9 and 13 after treatment, whereas anti-IgG2 was injected 24 h after the triple combination injection, at days 8 and 15. Tumour size was measured twice a week with an electronic calliper. Graphs reflect tumour growth profiles of mice after CD8 T lymphocytes depletion (*n* = 6/group). **e** Overall survival (*n* = 6/group) evaluated by log-rank test. ***p < 0.001 vs. control, ^###^*p* < 0.001 vs. triple therapy/α-IgG2.

In order to evaluate the quality of CD8^+^ T cell-dependent anti-tumour efficacy, the antigen specificity of tumour-infiltrating CD8^+^ T cells was assessed by E7 tetramer staining. The number of E7-specific CD8^+^ T lymphocytes increased in the tumour microenvironment with those therapies that increased the total number of CD8^+^ T lymphocytes: E7 long peptide, E7 long peptide/PIC and triple therapy (Fig. 5a). At day 13, all treatments increased the percentage of activated tumour-specific T lymphocytes, while only E7 long peptide/PIC and triple therapy reduced the percentage of exhausted tumour-specific T lymphocytes (Fig. 5b). The maximum and most homogenous increase was observed with triple therapy, as confirmed by immunohistochemistry staining. We observed a massive infiltration throughout the tumour bed of both CD3^+^ and CD8^+^ T lymphocytes 8 days after treatment (Fig. 5c). Depletion of CD8^+^ T cells abrogated the anti-tumour effect of the combination, demonstrating the essential role these cells play in the anti-tumour response of the triple therapy (Fig. 5d, e).

### Early effects of triple therapy on myeloid cells in the tumour microenvironment

**Fig. 6:**
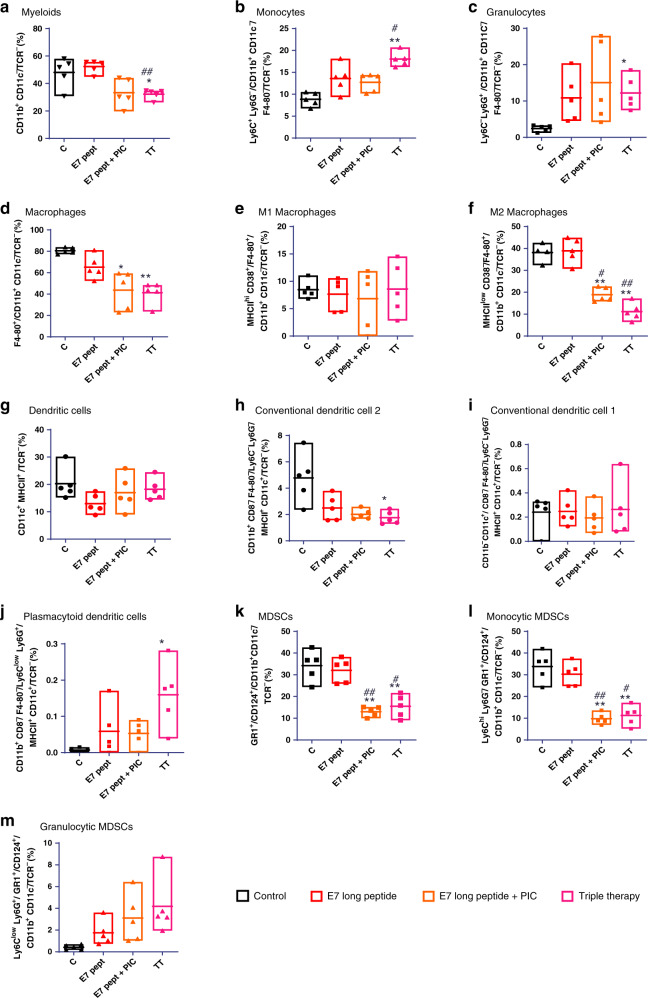
Characterisation of myeloid cell subpopulations in the tumour microenvironment 24 h after different combination immunotherapies. C57BL/6 mice were inoculated with 1 × 10^5^ TC-1/A9 cells, and 7 days later, they were treated with different combinations of an E7 long peptide (100 µg/mouse, i.t.), PIC (50 µg/mouse, i.t.) and anti-PD-L1 (200 µg/mouse, i.v.). One day after, mice were sacrificed and tumours collected for the analysis of the different myeloid subsets by flow cytometry (*n* = 5/group). **a** Myeloid cells (CD11b^+^CD11c^+^/TCR^−^). **b** Monocytes (Ly6C^+^Ly6G^−^/CD11b^+^CD11c^−^/F4-80^−^/TCR^−^). **c** Granulocytes (Ly6C^low^Ly6G^+^/CD11b^+^CD11c^−^/F4-80^−^/TCR^−^). **d** Macrophages (F4-80^+^/CD11b^+^CD11c^−^/TCR^−^). **e** M1 macrophages (MHCII^hi^CD38^+^/F4-80^+^/CD11b^+^CD11c^−^/TCR^−^). **f** M2 macrophages (MHCI^low^CD38^−^/Ly6C^−^Ly6G^−^/F4-80^+^/CD11b^+^CD11c^−^/TCR^−^). **g** Dendritic cells (CD11c^+^MHCII^+^/TCR^−^). **h** Conventional dendritic cells (CD11b^+^CD8^−^/F4-80^−^/Ly6C^−^Ly6G^−^/MHCII^+^CD11c^+^/TCR^−^). **i** Conventional dendritic cells 1 (CD11c^+^CD11b^−^/CD8^−^/F4-80^−^/Ly6C^−^Ly6G^-^/MHCII^+^CD11c^+^/TCR^−^). **j** Plasmacytoid dendritic cells (CD11b^+^CD8^−^/F4-80^−^/Ly6C^low^Ly6G^+^/MHCII^+^CD11c^+^/TCR^−^). **k** MDSCs (GR-1^+^/CD124^+^/CD11b^+^CD11c^−^/TCR^−^). **l** Monocytic MDSCs (Ly6C^hi^Ly6G^−^/GR-1^+^/CD124^+^/CD11b^+^CD11c^−^/TCR^−^). **m** Polymorphonuclear MDSCs (Ly6C^low^Ly6G^+^/GR-1^+^/CD124^+^/CD11b^+^CD11c^−^/TCR^−^). One-way ANOVA followed by Tukey’s multiple comparison tests. *p < 0.05 and **p < 0.01 vs. control; ^#^*p* < 0.05 and ^##^*p* < 0.01 vs. E7 long peptide.

Due to the relevance of myeloid cells in the therapeutic efficacy of the triple therapy, we performed a detailed characterisation of myeloid-derived cells at days 1, 3 and 13 after the administration of immunotherapy combinations that exerted an anti-tumour effect: E7 long peptide/PIC, E7 long peptide/anti-PD-1 and triple therapy. Figure S5 shows the gating strategy and Figs. 6 and S6 shows the different myeloid subpopulations at days 1, 3 and 13. Triple therapy induced an increase in proinflammatory monocytes (Figs. 6b and S6) and plasmacytoid dendritic cells (Fig. 6j). Other myeloid cell populations associated with a dampened anti-tumour immune response decreased after triple treatment. This was particularly relevant for M2 tumour-associated macrophages (Fig. 6f) and myeloid-derived suppressor cells (Fig. 6k). Therefore, all these data together suggest that the triple therapy is characterised by an early remodelling of the tumour-associated myeloid cell compartment. The combined activity of E7 long peptide, PIC and anti-PD-1 is required for this remodelling to occur.

## Discussion

The clinical success of IC inhibitors relies on the unprecedented long-lasting responses achieved in a fraction of patients with certain tumours, such as melanoma, kidney cancer, non-small cell lung cancer and other immune-sensitive tumours.^26,27^ To improve the clinical outcome of IC inhibitors, multiple combinations are being tested in clinical trials. In September 2019, 2975 combination clinical trials were active worldwide.^21^ As a result of this extensive clinical research, some combinations have gained approval by regulatory agencies.^28–32^ The design of these combination trials will benefit from the development of mechanistic computational models that integrate the dynamics of immune cells promoted by the treatments.^33–35^ The building of these complex models requires longitudinal data on the anti-tumour effect and effector immune cells. Thus, we designed this study to gain insight into the immune dynamics in the tumour microenvironment that characterise successful immunotherapy combinations. To achieve different anti-tumour efficacy rates, we combined the i.t. administration of the TC-1/A9-immunodominant E7 epitope in the form of a long peptide and/or the TLR-3-ligand PIC with systemic administration of the IC inhibitor anti-PD-1 mAb. Intratumoural immunotherapy can achieve a high local concentration of the immunostimulatory compounds, minimising systemic exposure and, therefore, immune-related adverse effects.^36^ This strategy is being pursued in clinical trials with several compounds that activate pattern recognition receptors such as TLR ligands and viruses, but intratumoural vaccination has also demonstrated clinical advantages.^37–40^ Direct inoculation of the vaccine into the tumour bed improves the activation and antigen uptake by Batf3-dependent dendritic cells, a subset of antigen-presenting cells critical for the activation of an anti-tumour immune response due to their ability to produce high levels of IL-12 in the tumour microenvironment and to cross-present tumour-associated antigens.^41,42^ In our experimental setting, the intratumoural administration of the E7 long peptide was the key immunostimulatory compound. The priming effect was triggered by the peptide alone or the peptide combined with PIC, upregulated proinflammatory markers in myeloid cells and increased the early infiltration of CD8^+^ T lymphocytes. In contrast, PIC or anti-PD-1 as monotherapy or the combination of both immunostimulatory compounds only modulated myeloid cells, but did not have any impact on intratumoural CD8^+^ T cells. Moreover, PIC markedly upregulated Treg at the latest time point. Consistent with our data, a previous study showed high levels of IL-10 production in 24 h cultures of splenocytes exposed to PIC, a phenomenon that was associated with a limited expansion and function of effector CD8^+^ T cells in a bacterial infection mouse model.^43^ The immunomodulatory effects exerted by the combination of the E7 long peptide with PIC were enhanced by the systemic administration of anti-PD-1. This triple therapy was extremely efficient at remodelling the myeloid cell compartment as early as 24 h after treatment initiation. Proinflammatory monocytes, characterised by the expression of Ly6C, were markedly increased while immunosuppressive myeloid subsets such as M2 macrophages and myeloid-derived suppressor cells decreased. This modulation of the myeloid cell compartment may be due to the release of proinflammatory cytokines in the tumour microenvironment,^25,44^ but also supports the effect of anti-PD-1 on myeloid cells as has been recently demonstrated by genetic deletion of PD-1 in myeloid cells.^45^ Recently, high serum levels of IL-8 released by myeloid cells in the tumour microenvironment have been shown to correlate with reduced clinical benefit to anti-PD-1/PD-L1 mAbs.^46,47^ Our data indicate that the deleterious effect of myeloid cells on IC blockade anti-tumour efficacy can be overcome by intratumoural administration of immunostimulatory agents. Modulation of myeloid cells may be superior to other strategies in clinical development aimed at depleting myeloid cells since the early modulation of myeloid cells preceded the sustained infiltration of CD8^+^ and CD4^+^ T lymphocytes, which was essential for anti-tumour efficacy. This dynamic behaviour of the critical immune subset highlights the need for preclinical-driven longitudinal sampling in biomarker studies in clinical trials. The integration of longitudinal preclinical data into mathematical computational models will help to guide new clinical trial designs.

In conclusion, the analysis of the kinetics of the immune response induced by immunotherapies with different anti-tumour efficacies has revealed the critical role of the early modulation of the myeloid cell compartment to allow subsequent infiltration of effector T lymphocytes, thus highlighting the relevance of the myeloid subset as an interesting but dynamic biomarker for immunotherapies.

## Supplementary information

Supplementar material

## Data Availability

The datasets used during the current study are available from the corresponding author on reasonable request.
