# Supplementary material for: Modulation of intratumoural myeloid cells, the hallmark of the anti-tumour efficacy induced by a triple combination: tumour-associated peptide, TLR-3 ligand and α-PD-1
Source: Br J Cancer. 2021 Feb 3;124(7):1275–85. doi: 10.1038/s41416-020-01239-z (PMC8007692; doi:10.1038/s41416-020-01239-z)
Supplement: Supplementary file 1 — Supplementar material [file 41416_2020_1239_MOESM1_ESM.docx]

**Supplementary material**

***Table S1****. List of antibodies used.*

| Antibody | Color | Clone | Dilution | Specification | Provider |
| --- | --- | --- | --- | --- | --- |
| CD11b | APC | M1/70 | 1:200 | Cytometry | BioLegend |
| CD11b | BUV395 | M1/70 | 1:200 | Cytometry | BD Bioscience |
| Ly6c | PerCy5 | RB6-8C5 | 1:400 | Cytometry | BioLegend |
| Ly6c | FITC | AL-21 | 1:400 | Cytometry | BD Bioscience |
| PD-L1 | BV421 | 10F.9G2 | 1:200 | Cytometry | BioLegend |
| PD-L1 | PERCPCy5.5 | 10F.9G2 | 1:200 | Cytometry | BioLegend |
| PD-L1 | n.s. | D5V3B | n.s. | IHC | Cell Signalling |
| CD8 | BV510 | 56-6.7 | 1:200 | Cytometry | BioLegend |
| CD8 | PECy7 | 53-6.7 | 1:200 | Cytometry | BioLegend |
| CD8 | BUV395 | 53-6.7 | 1:200 | Cytometry | BD Bioscience |
| CD8 | n.s. | 53-6.7 | n.s. | ICH | BD Bioscience |
| F4/80 | FITC | BM8 | 1:200 | Cytometry | BioLegend |
| F4/80 | AF647 | BM8 | 1:200 | Cytometry | BioLegend |
| CD124 | PE | I015F8 | 1:200 | Cytometry | BioLegend |
| Ly6g | BV510 | 1A8 | 1:200 | Cytometry | BioLegend |
| CD11c | PeCy7 | N418 | 1:200 | Cytometry | BioLegend |
| CD11c | BV650 | N418 | 1:200 | Cytometry | BioLegend |
| CD45 | BV510 | 30-F11 | 1:400 | Cytometry | BioLegend |
| CD45 | BV421 | 30-F11 | 1:200 | Cytometry | BioLegend |
| TCRβ | BV605 | H57-597 | 1:200 | Cytometry | BioLegend |
| TCRβ | BV785 | H57-597 | 1:200 | Cytometry | BioLegend |
| CD19 | BV650 | 6D5 | 1:200 | Cytometry | BioLegend |
| MHCII | APCCy7 | M5/114.15.2 | 1:400 | Cytometry | BioLegend |
| CD38 | PE-Dazzle594 | 90 | 1:100 | Cytometry | BioLegend |
| CD16/32 | n.s. | 2.4G2 | 1:200 | Cytometry | BD Bioscience |
| CD4 | BUV496 | GK1.5 | 1:400 | Cytometry | BD Bioscience |
| E7tetramer | PE | n.s. | 1:50 | Cytometry | Immudex |
| α-rat-IgG | AF488 | A21210 | 1:400 | Cytometry | Molecular Probes |
| CD3 | n.s. | SP7 | n.s. | ICH | Thermofisher |
| PD-1 | BV785 | 29F.1A12 | 1:100 | Cytometry | BioLegend |
| Tim3 | Pe-Dazzle594 | B8.2C12 | 1:80 | Cytometry | BioLegend |

ICH: Immunohistochemistry

***Figure S1****. Schematic representation of experimental protocols. a) Dosing schedule for the different treatment combinations. b) Administration of the different components of monotherapies, bitherapies, and triple therapy over time.*

*Peptide: E7 long peptide; α-PD-1: Anti-PD-1; PIC: polyinosinic–polycytidylic acid.*

***Figure S2.*** *Experimental design of longitudinal biomarkers evaluation. A total of 72 tumor-bearing mice divided into eight groups were used in this experiment. Treatments started at day 7 after tumor cell inoculation, and at 3, 8, and 13 post-dosing days, tumors corresponding to 3 mice per group were collected (n=24 mice/time). MT: monotherapy; BT: bitherapy; TT: triple therapy.*

*
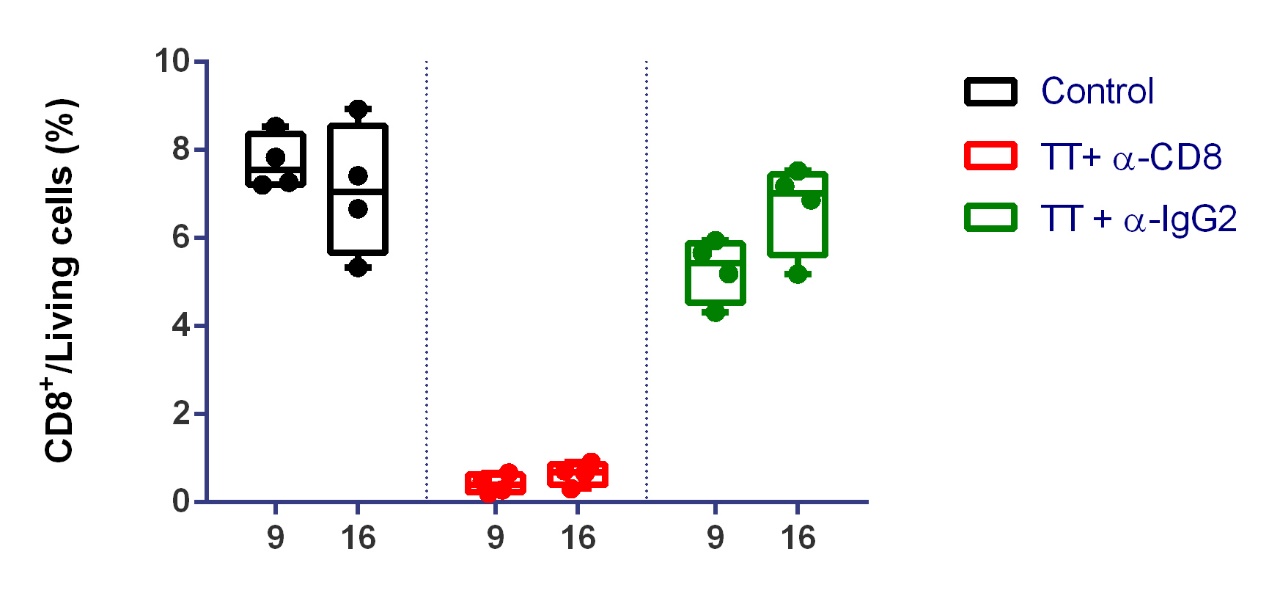
*

***Figure S3****. Evaluation of CD8^+^ depletion. At day 7 after 1x10^5^ TC-1/A9 cell inoculation, mice were divided into three groups: Control, Triple therapy combined with anti-CD8 antibody, and Triple therapy combined with the irrelevant polyclonal rat anti-IgG2. Doses of 200 µg/mouse of anti-CD8 mAb were i.p. administered 24 h before therapeutic treatments and at days 2, 6, 9, and 13 after treatment; whereas anti-IgG2 was injected 24 h after the triple combination injection, at days 8 and 15. The percentage of CD8^+^ T lymphocytes in circulation analyzed by flow cytometry is shown at days 9 and 16 after TT treatment. TT: Triple therapy.*

***Figure S4****. Evaluation of immune memory effect. C57BL/6 mice were inoculated with 1x10^5^ TC-1/A9 cells, and 7 day later, mice were treated with E7 long peptide (100 µg/mouse, it) and PIC (50 µg/mouse, it) and α-PD-1 (200 µg/mouse, iv). Tumor growth profiles were followed for 90 days. Survival mice (2 in the case of bitherapies and 3 in the case of the triple combination) were administered with 1x10^5^ TC-1/A9 cells as a rechallenge (black arrows). As control, three naïve mice were administered with 1x10^5^ TC-1/A9 cells at the same time point. The experiment was finished when the control mice were sacrificed.*


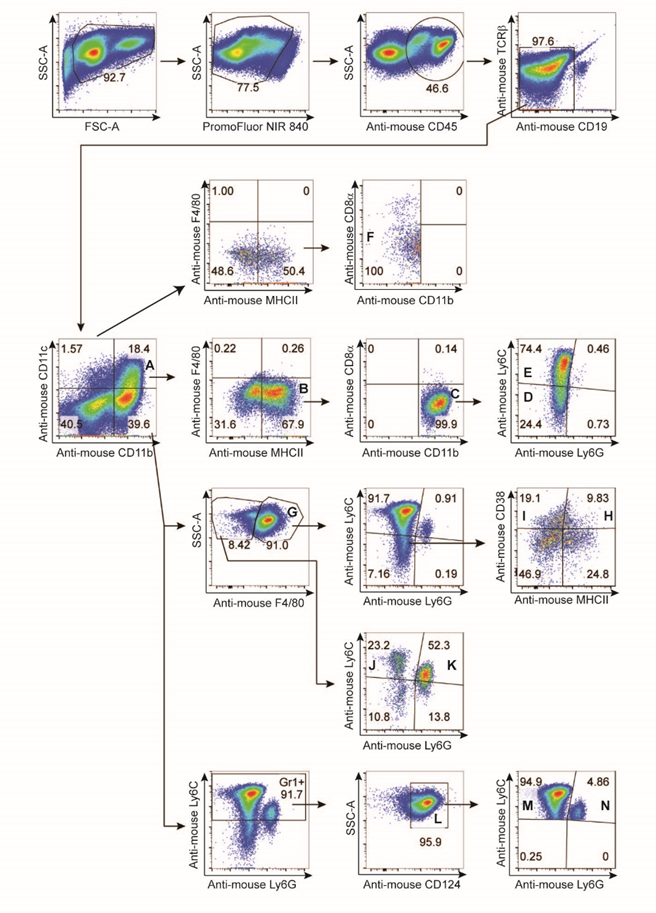


***Figure S5****. Schematic representation of gating strategies to define the myeloid compartment in tumor samples. Cells first were selected for TCR^-^ cells by gating in live scatter, live cells (PromoFluor NIR 840^-^) CD45^+^, CD19^-^ and TCRb^-^. A) Myeloid cells (CD11b^+^CD11c^+^/TCR^-^); B) Dendritic cells (CD11c^+^MHCII^+^/TCR^-^); C) cDC2 (CD11c^+^MHCII^+^/F4-80^-^CD11b^+^CD8^-^/TCR^-^); D) Conventional dendritic cells (CD11b^+^CD8^-^/F4-80^-^/Ly6C^-^Ly6G^-^/MHCII^+^CD11c^+^/TCR^-^); E) Plasmacytoid dendritic cells (CD11b^+^CD8^-^/F4-80^-^/Ly6C^hi^Ly6G^-^/MHCII^+^CD11c^+^/TCR^-^); F) cDC1 (CD11c^+^MHCII^+^/F4-80^-^CD11b^--^/TCR^-^); G) Macrophages (F4-80^+^/CD11b^+^CD11c^-^/TCR^-^); H) M1 Macrophages (MHCII^hi^CD38^+^/Ly6C^-^Ly6G^-^/F4-80^+^/CD11b^+^CD11c^-^/TCR^-^); I) M2 Macrophages (MHCI^low^CD38^-^/F4-80^+^/CD11b^+^CD11c^-^/TCR^-^); J) Monocytes (Ly6C^+^Ly6G^-^/CD11b^+^CD11c^-^/F4-80^-^/TCR^-^); K) Granulocytes (Ly6C^low^Ly6G^+^/CD11b^+^CD11c^-^/F4-80^-^/TCR^-^); L) MDSCs (GR1^+^/CD124^+^/CD11b^+^CD11c^-^/TCR^-^); M) Monocytic MDSCs (Ly6C^hi^Ly6G^-^/GR1^+^/CD124^+^/CD11b^+^CD11c^-^/TCR^-^); N) Polymorphonuclear MDSCs (Ly6C^low^Ly6G^+^/GR1^+^/CD124^+^/CD11b^+^CD11c^-^/TCR^-^). Data shows representative dot-plots of a sample.*


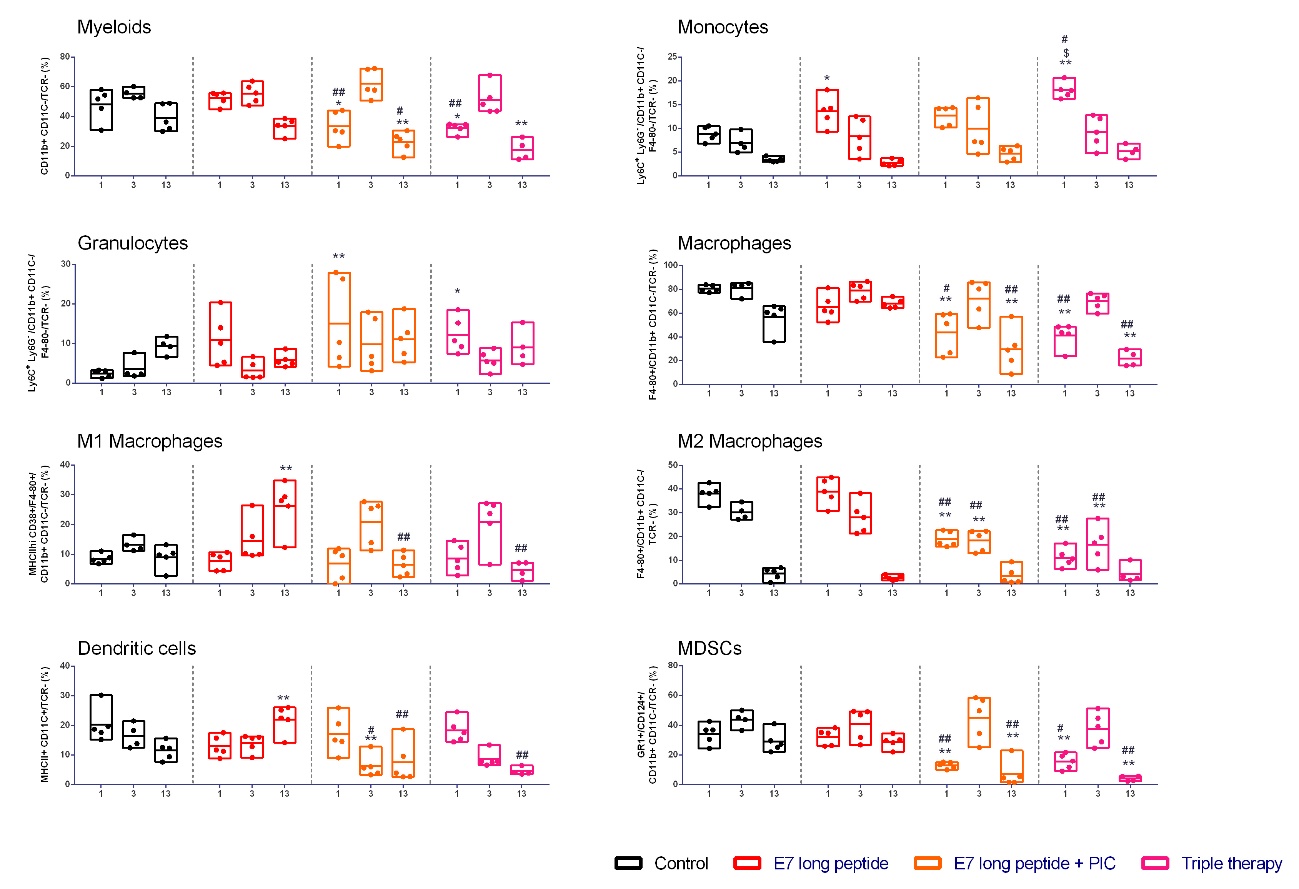


***Figure S6****. Longitudinal analysis of main myeloid cell subpopulations in tumor tissue at days 1, 3, and 13. C57BL/6 mice were inoculated with 1x10^5^ TC-1/A9 cells, and 7 days later, they were treated with different combinations of an E7 long peptide (100 µg/mouse, it), E7 long peptide/PIC (100 and 50 µg/mouse, it), and Triple therapy. At days 1, 3, and 13 after treatment administration, mice were sacrificed, and tumors were collected to analyze the different myeloid subsets by flow cytometry (n=5/group).* p< 0.05 and ** p<0.01 vs control, # p<0.05 and ## p<0.01 vs peptide, $ p< 0.05 and $$ p< 0.01 vs bitherapy.*
